# High estradiol levels during a long agonist IVF protocol are associated with decreased food intake, higher leptin concentrations, and lower levels of high-sensitivity C-reactive protein

**DOI:** 10.1007/s00404-023-06950-9

**Published:** 2023-02-16

**Authors:** Jonna Leppänen, Petrus Nuotio, Kaisa Randell, Jarkko Romppanen, Leea Keski-Nisula, Tomi Laitinen, Jussi Pihlajamäki, Ursula Schwab, Seppo Heinonen

**Affiliations:** 1 Department of Obstetrics and Gynecology, Kuopio University Hospital and University of Eastern Finland, Puijonlaaksontie 2, 70210 Kuopio, Finland; 2 Institute of Public Health and Clinical Nutrition, University of Eastern Finland, Kuopio Campus, P.O. Box 1627, 70211 Kuopio, Finland; 3 Pihlajalinna Dextra Fertility Clinic, Helsinki, Finland; 4 Eastern Finland Laboratory Centre, Puijonlaaksontie 2, 70210 Kuopio, Finland; 5 Department of Medicine, Endocrinology and Clinical Nutrition, Kuopio University Hospital, Puijonlaaksontie 2, 70210 Kuopio, Finland; 6 Department of Clinical Physiology and Nuclear Medicine, Kuopio University Hospital and University of Eastern Finland, Puijonlaaksontie 2, 70210 Kuopio, Finland; 7 Obstetrics and Gynecology, University of Helsinki and Helsinki University Hospital, PO Box 140, 00029 Helsinki, Finland

**Keywords:** Infertility, In vitro fertilization, Appetite regulation, Estradiol, Leptin

## Abstract

**Purpose:**

To study whether different hormonal phases affect appetite regulation, food intake, and concentrations of leptin, glucagon-like peptide-1 (GLP-1), and high-sensitivity C-reactive protein (hs-CRP) during a long agonist in vitro fertilization (IVF) protocol.

**Methods:**

Fifty-four infertile women were encountered thrice, the first of which was at the beginning of their period (low estradiol). The other two visits were during a gonadotrophin-releasing hormone (GnRH) analog downregulation (low estradiol) and at the end of a follicle-stimulating hormone (FSH) stimulation (high estradiol). The first visit was the reference; the women served as their controls. The concentrations of leptin, GLP-1, and hs-CRP were assessed from plasma. Dietary intake was assessed using food records (FRs). In addition, weight, height, body mass index (BMI), and plasma levels of estradiol, glucose, HbA1c, insulin, and lipids were monitored. Twenty-six of the subjects also had a postprandial test.

**Results:**

During the stimulation protocol, leptin concentrations elevated (*P* < 0.001), and energy intake decreased (*P* = 0.03), while estradiol levels increased (*P* < 0.001). GLP-1 levels unchanged (*P* = 0.75) and hs-CRP (*P* = 0.03) concentrations diminished, while estradiol levels increased.

**Conclusion:**

No increased food intake or weight gain occurred during the stimulation protocol; thus, leptin may protect from overeating during high estradiol levels, and leptin resistance may not occur during a short follow-up. Also, a favorable anti-inflammatory effect was detected. During this study, we observed no harmful metabolic effects, which might affect negatively maternal health.

## What does this study add to the clinical work

**Table:** 

No increased food intake or weight gain occurred during the long agonist IVF protocol, and a beneficial anti-inflammatory effect was noticed. Thus, no harmful metabolic effects were observed, which might negatively affect maternal health.	

## Introduction

Sex hormones have essential roles in regulating appetite, food intake, and energy metabolism by interacting with gastrointestinal peptides, neurotransmitters, and adipocytes [1, 2]. Estrogen inhibits food intake, but in the presence of estrogen, progesterone enhances appetite and promotes weight gain (2). Thus, food intake varies during the menstrual cycle [2]. A mean food intake is reduced during the periovulatory phase of the menstrual cycle when estradiol levels are high [3, 4].

Adipocytes produce leptin. It inhibits food intake and involves energy expenditure, storage of fat, and insulin signaling [5]. When bodyweight is steady, leptin levels are an indicator of body fat mass. In turn, during weight loss or weight gain phases, leptin signals for an energy imbalance. Leptin informs the central nervous system about the abundance and availability of energy deposits [6]. Decreased leptin concentrations induce hunger, whereas high leptin concentrations in obese individuals due to chronic overfeed lead to leptin resistance [5].

Glucagon-like peptide-1 (GLP-1) is an anorexigenic peptide hormone released in response to a meal from the intestinal mucosa enteroendocrine cells. It is one of the gut peptides that increases satiety and suppresses appetite in normal-weight individuals [7]. GLP-1 receptors are located in the central nervous system and the periphery [8, 9]. In addition, GLP-1 stimulates insulin release in pancreatic ß-cells, thus also regulating glucose homeostasis [7]. GLP-1 concentrations are low at fasting conditions; however, increasing rapidly after eating fat or carbohydrates. Hence, GLP-1 regulates food intake, and the administration of exogenous GLP-1 or its long-acting analogs reduces food consumption [9].

Hs-CRP is a marker of low-grade inflammation, and it is chronically elevated in patients with atherosclerosis and obesity [10]. It is a robust independent predictor of future atherothrombotic events among people without known cardiovascular disease [10]. Hs-CRP facilitates the adhesion and migration of monocytes into the arterial wall, and it also has an inhibitory effect on nitric oxide synthesis following altered vascular reactivity [6].

Estradiol levels are deficient at the beginning of a menstrual cycle and the beginning of a long agonist IVF protocol when patients are using GnRH-agonist medication. After that period, patients start FSH stimulation and estradiol levels increase. Estrogen concentrations may be tenfold greater than levels during the ovulatory phase in a normal menstrual cycle.

Our study aimed to examine appetite regulation, food intake, and release of leptin, GLP-1, and hs-CRP in different hormonal phases during a long agonist IVF protocol.

## Methods

### Participants

We studied 54 infertile women, ranging in age from 24 to 40 years (33 ± 4 years, mean ± SD), who attended a long agonist IVF protocol in the Kuopio University Hospital infertility clinic. Subjects were recruited from the infertility clinic while meeting the doctor for IVF treatment planning. Twenty-six of the subjects participated in a postprandial test, and the rest twenty-eight patients had only fasting blood samples of the different variables.

### Study protocol and meal

The subjects were encountered thrice. The first time (Visit 1) was at the beginning of the menstrual cycle without any hormonal treatment (from the second to the fifth days of the period). Then, agonist medication (GnRH-analog, nafarelin acetate 800 mcg/day) was initiated approximately one week before the next period. The patient came to the subsequent control (Visit 2) about one month after Visit 1. Then, patient's estradiol level was supposed to be very low, like in postmenopausal level. In Visit 2, subjects started the controlled ovarian stimulation with FSH. The FSH dose varied from 125 to 300 IU, and it was adapted to the patient's body mass index (BMI), ovarian reserve, and age. In Visit 3, subjects met the doctor for the third time after administering FSH daily for nine days. Then, the estradiol levels were supposed to be increased compared to periovulatory levels during a normal menstrual cycle. Blood samples and clinical measurements were obtained at every three visits. For the hormonal intervention, primary outcome variables were the concentrations of leptin, GLP-1, and hs-CRP.

A postprandial test was performed for 26 of the 54 patients. First, fasting samples were taken (0 min) and then subjects ate a standard breakfast (185 g (g) of porridge, 150 g yogurt, and a glass of water, a total of 266 kcal). Next, they had a cannula in the antecubital vein, and a nurse took blood samples at 30, 60, 120, and 240 min (min). At all three visits, plasma levels of leptin, GLP-1, and hs-CRP were determined at those five time points.

Secondary outcomes were serum levels of glucose, HbA1c, insulin, estradiol, and plasma lipid levels. If data from any of the three visits were missing, the patient was excluded from the study, except levels of glucose and HbA1c (*n* = 53), insulin (*n* = 48), and leptin (*n* = 44).

Height and weight were measured at each visit, and BMI was calculated. All laboratory tests and data on appetite profile and dietary intake were blindly analyzed by personnel who had no information of the IVF cycle phase.

### Appetite profile and dietary intake

Dietary intake was assessed using food records (FRs), in which food and beverage intakes were reported for three consecutive days before each study visit. Participants who failed to fill FRs beforehand were advised to fill their FR on the days following the study visit. Portion sizes were weighed or estimated using household measures. Thirteen participants who failed to fill all three FRs were excluded from the nutrient intake analyses. Nutrient intake from the diet (i.e., vitamin and mineral supplements excluded) was calculated using AivoDiet software (version 2.2.0.0., AivoFinland Oy, Turku, Finland) based on national and international analyses, and international food composition tables (fineli.fi).

### Blood samples

Overnight fasting blood samples (12 h) were taken and analyzed in the laboratory. Samples were centrifuged at 2000 rpm for 10 min, and serum plasma was separated. All lipid, estradiol, and hs-CRP determinations were analyzed using standard methods, as previously described [11, 12]. Total GLP-1 was analyzed with Cloud-Clone Corp., ELISA (LOT: L190104265, Exp. Sep. 2019), with a working range of 12.35 pg/ml–1000 pg/ml. Leptin was determined with R&D systems (a Bio-techne brand, Quantikine ELISA. Catalog No. DLP00, LOT: P221489, Exp. 03 Nov 2020), with measurement lower boundary under 7.8 pg/ml. Glucose was analyzed by enzymatic method with hexokinase using Cobas 6000 (c 501) analyzer (Hitachi High Technology Co, Tokyo, Japan). Reagent assessed was Glucose HK (GLUC3), (cat. nro 04404483 190, Roche Diagnostics GmbH, Mannheim, Germany. ACN 717). The measuring range was 0.1–41.6 mmol/l. HbA1c was determined by Turbidimetric inhibition immunoassay (TINIA) with Cobas 6000-analyzer (Hitachi High Technology Co, Tokyo, Japan). Reagents used: 1. Tina-quant Hemoglobin A1c Gen.3 (A1C-3), (Cat. no. 05336163 190, Cobas c systems, Roche Diagnostics GmbH, Mannheim, Germany), 2. Hemolyzing Reagent Gen.2 (A1CD2), (Cat. No. 04528182 190, Cobas c systems, Roche Diagnostics GmbH, Mannheim, Germany), and 3. Special Cell Cleaning Solution (SCCS), (Cat. No. 04880994 190, Cobas c systems, Roche Diagnostics GmbH, Mannheim, Germany). Measuring range was Hb 40–400 g/l, HbA1c 3.0–26.0 g/l, unit mmol/mol. Insulin was assessed by ECLIA with a Cobas e 601-analyzer (Hitachi High Technology Co, Tokyo, Japan). Reagent assessed was Insulin, (cat nro 12017547 122, Roche Diagnostics GmbH, Mannheim, Germany). The measuring range was 0.2–1000 mU/l.

### Statistical analyses

Statistical analyses and calculations were done with IBM SPSS Statistics (version 27 for Macintosh, Armonk, NY.) and R (version 4.2.1). Descriptive data are expressed as means ± SD in tables and as means (95% Confidence intervals) in figures. Kolmogorov–Smirnov test was performed to test distribution's normality. This test exhibited skewed distributions in main variables, and statistical analyses were accomplished with non-parametrical tests. Friedman’s test was used to detect the difference in the values between separate time points. If the significance was observed between time points, Wilcoxon's test analyzed differences between two time points. The responses to the postprandial tests were compared between the study visits using repeated-measures ANOVA, with log10-transformed values of either leptin, GLP-1 or hs-CRP concentration as the dependent variable and postprandial time point and study visit as within-subjects factors. Univariate correlations were defined using Spearman correlation. *P* < 0.05 was considered statistically significant.

Dietary data were analyzed using IBM SPSS Statistics (version 27, IBM inc., Armonk, New York) and R (version 4.2.1). Shapiro–Wilk test was used to assess the normality of the data. Non-normally distributed variables were log10-transformed. Repeated-measures ANOVA and Friedman test were used for normally and non-normally distributed variables, respectively. Bonferroni correction was applied to all post hoc tests. A two-tailed *P*-value of < 0.05 was considered as statistically significant.

## Results

The clinical characteristics in Visits 1, 2 and 3, and responses to a long agonist IVF protocol are shown in Table 1.

**Table 1:** Clinical characteristics and responses to a long agonist IVF protocol

	Visit 1	Visit 2	Visit 3	*P*-value
Age (years)	33 ± 4	–	–	–
Gravity	0.6 ± 0.9	–	–	–
Para	0.3 ± 0.5	–	–	–
Height (cm)	166 ± 5	–	–	–
Weight (kg)	69.9 ± 14.3	69.9 ± 14.1	69.7 ± 13.8	–
Body mass index (kg/m^2^)	25.3 ± 5.2	25.3 ± 5.1	25.3 ± 5	–
Glucagon-like peptide-1 (pg/ml)	34.1 ± 5.8	33 ± 5.4	33.1 ± 5.6	NS
Leptin (pg/ml)^a^	17,887.3	16113.4	21356	< 0.001
	± 13661.2^#^	± 14397.3§	± 16038.2	
High-sensitive C-reactive protein (mg/l)	3.5 ± 8*^#^	2.3 ± 5.1	1.8 ± 3.5	0.03
Insulin (mU/l)^b^	6.9 ± 3	7 ± 4.7	8.2 ± 7.3	NS
Glucose (mmol/l)^c^	5.3 ± 0.4#	5.3 ± 0.4	5.1 ± 0.4¤	0.002
Hemoglobin A1c (mmol/mol)^c^	32.9 ± 2.5	32.8 ± 2.8	32.6 ± 2.7	NS
Total cholesterol (mmol/l)	4.7 ± 0.8*^†^	4.9 ± 0.8^§^	4.5 ± 0.7	< 0.001
LDL cholesterol (mmol/l)	2.8 ± 0.7*^†^	2.9 ± 0.7^§^	2.5 ± 0.7	< 0.001
HDL cholesterol (mmol/l)	1.7 ± 0.3	1.7 ± 0.3	1.7 ± 0.3	NS
Triglycerides (mmol/l)	0.8 ± 0.3	0.8 ± 0.3	0.8 ± 0.3	NS
Total/HDL cholesterol	2.9 ± 0.8†	3 ± 0.7^§^	2.7 ± 0.7	< 0.001
Estradiol (nmol/l)	0.2 ± 0.1^‡†^	0.1 ± 0.1^§^	6.4 ± 5.5	< 0.001
Estradiol (nmol/l)•	0.04–0.75	0.04–0.61	0.63–31.5	

The values are means ± SD

•,*P < 0.05 Visit 1 vs. Visit 2

^#^*P* < 0.05 Visit 1 vs. Visit 3

^§^*P* < 0.001 Visit 2 vs. Visit 3

¤*P* < 0.05 Visit 2 vs. Visit 3

^†^*P* < 0.001 Visit 1 vs. Visit 3

^‡^*P* < 0.001 Visit 1 vs. Visit 2

•A range of estradiol values

^a^*n* = 44

^b^*n* = 48

^c^*n* = 53

**Fig. 1:**
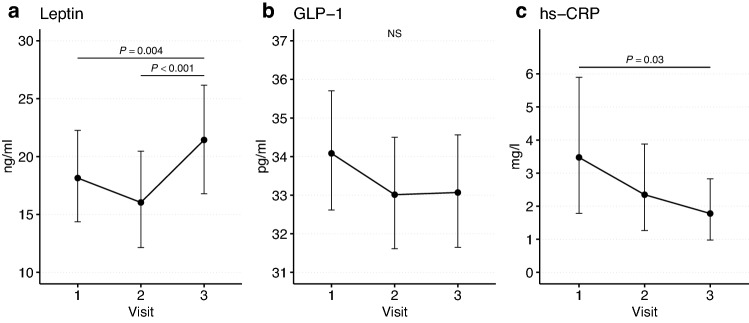
Fasting plasma concentrations of leptin (**a**), glucagon-like peptide-1 (GPL-1, **b**), and high-sensitivity CRP (hs-CRP, **c**) at three visits. Points and error bars indicate means and their 95% confidence intervals

The values in leptin, GLP-1, and hs-CRP at three visits are shown in Fig. 1. Leptin levels varied significantly during the IVF stimulation, and those levels were at the lowest in Visit 2. The highest leptin levels were reached at the third Visit. The changes in GLP-1 values were statistically non-significant. The values in hs-CRP declined significantly throughout the protocol from Visit 1 to Visit 3.

The changes in insulin and HbA1c levels were non-significant (Table 1). Fasting glucose levels diminished between Visits 2 and 3 (Table 1).

Lipid profile changed through Visits 1, 2, and 3 (Table 1). Total and LDL cholesterol concentrations decreased towards the end of stimulation, and also, the total/HDL cholesterol ratio diminished between Visits 2 and 3. However, HDL cholesterol and triglyceride level were unchanged significantly.

**Fig. 2:**
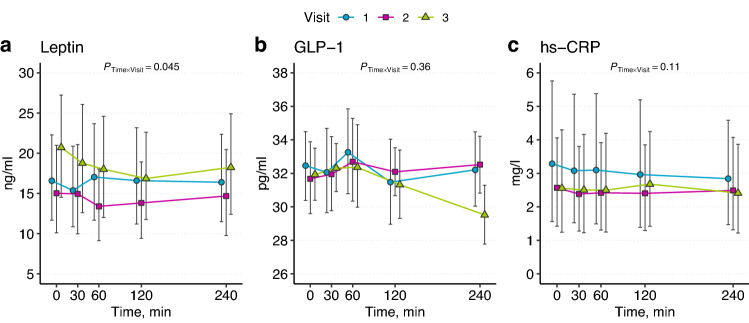
Changes in plasma concentrations of leptin (**a**), glucagon-like peptide-1 (GPL-1, **b**), and high-sensitivity CRP (hs-CRP, **c**) in response to the postprandial test at three Visits. Points and error bars indicate means and their 95% confidence intervals

The values of leptin, GLP-1, and hs-CRP during a postprandial test in three visits are presented in Fig. 2.

The changes in GLP-1 levels in three visits were non-significant.

The leptin values were analyzed from 19 subjects at each Visit for the postprandial test since some values were missing. Leptin levels were unchanged in Visit 1. In Visits 2 and 3, leptin values changed significantly. The most notable change in leptin values in Visit 2 was between 30 and 60 min (*P* = 0.02), and in Visit 3, between 0 and 30 min (*P* = 0.04).

The hs-CRP values decreased in Visits 1 and 2, but at the third Visit, the change was non-significant. The most notable change in hs-CRP levels in Visit 1 was between 0 and 30 min (*P* = 0.002) and in Visit 2 between 0 and 240 min (*P* < 0.001).

**Fig. 3:**
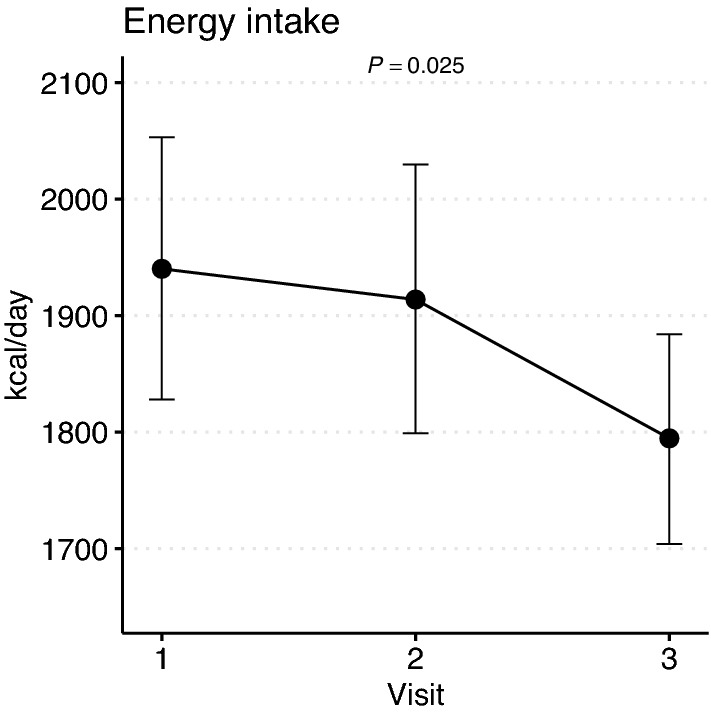
The changes in energy intake in three visits. Points and error bars indicate means and their 95% Confidence Intervals

The changes in nutrient intake are presented in Table 2 and Fig. 3. The energy intake differed significantly between Visits 1 and 3, and it was at the lowest during the third Visit. The intake of energy nutrients and fiber were unchanged between the three visits. Totally 21 patients reported using alcohol during stimulation, and intake diminished significantly during three visits (*P* = 0.02), and only seven persons reported using alcohol at Visit 3.

**Table 2:** Changes in nutrient intake based on 3-day dietary records (*n* = 52)

	Mean ± SD or median (Q1; Q3)			*p*-value	*p*-value of post hoc tests^a^		
Visit(s)	1	2	3		1 vs. 2	2 vs. 3	1 vs. 3
Energy (kcal)	1940 ± 422	1914 ± 426	1795 ± 337	**0.025**	1.000	0.172	**0.026**
Protein (*E*%)^†^	18.1 ± 4.0	18.1 ± 3.6	17.8 ± 3.8	0.690	–	–	–
Carbohydrate (*E*%)	43.8 ± 6.7	43.3 ± 7.1	44.2 ± 6.9	0.688	–	–	–
Total fat (*E*%)	35.3 ± 5.9	35.5 ± 7.3	35.4 ± 6.9	0.977	–	–	–
UFA (% of fat)^‡^	57.0 ± 6.2	56.1 ± 6.7	57.5 ± 7.4	0.584	–	–	–
Cholesterol (mg)^†^	232 ± 88	246 ± 104	242 ± 100	0.742	–	–	–
Fiber (g)	21.8 ± 6.9	20.6 ± 6.9	19.7 ± 6.3	0.103	–	–	–

Bold values indicate *p*-value < 0.05 was considered statistically significant

*UFA* unsaturated fatty acids

^†^Variable has been log_10_-transformed

^‡^Friedman test was used

^a^Bonferroni-corrected pairwise comparison between visits

The GLP-1 values correlated only with HbA1c in Visit 3 (*r* = 0.279, *P* = 0.04). No correlations between GLP-1 and other variables existed.

Statistically significant correlations between leptin and hs-CRP and different variables are presented in Table 3.

**Table 3:** Statistically significant correlations between leptin and/or hs-CRP and selected variables in Visits 1, 2, and 3. Spearman correlation coefficient was used

	Leptin			hs-CRP		
	1	2	3	1	2	3
	*r*	*r*	*r*	*r*	*r*	*r*
Insulin (mU/l)	0.494^¤^	0.331*	0.482^¤^			− 0.332*
Total cholesterol (mmol/l)	0.310*	0.290*	0.407*			
Low-density lipoprotein (mmol/l)	0.343*	0.398*	0.462**			
High-density lipoprotein (mmol/l)			− 0.272^#^		− 0.364*	− 0.332*
Triglycerides (mmol/l)	0.469^¤^	0.471**	0.589**		0.379*	0.447^¤^
Weight (kg)	0.685**	0.735**	0.668**	0.429^¤^	0.393*	0.514**
Leptin (pg/ml)				0.505**	0.426*	0.487**

^¤^*P* = 0.001

*P < 0.05

**P < 0.001

^#^*P* = 0.001

## Discussion

We found that during the stimulation phase of a long agonist IVF protocol, when the estradiol level was low and then increased, leptin concentrations peaked, and energy intake decreased. GLP-1 values unchanged during a long agonist protocol. Toward the end of stimulation, hs-CRP concentrations decreased. During a postprandial test, GLP-1 levels remained. Leptin values varied significantly during low and high estradiol levels. Hs-CRP values descended during the low estradiol levels.

Our findings are novel, with no earlier studies about appetite regulation and food intake during a long agonist IVF protocol. Therefore, we had to compare our results to other hormonal statuses when estradiol levels are high or low. Hormonal fluctuations during the menstrual cycle influence appetite control and food intake [4, 13]. A mean food intake decreases at the periovulatory phase with high estradiol levels [2]. That finding is similar to our results.

An increase in food intake and appetite control occurs during pregnancy to verify fetuses’ normal development and growth [2]. The increase of food intake in humans is 10–15% [14]. However, this enhancement of food intake remains elucidated, but sex hormones, especially progesterone, are probably involved [15]. Also, leptin levels increase during pregnancy, and resistance to central anorectic actions of leptin occurs [16]. In contrast, we found an increase in leptin levels and a decrease in energy intake during the higher estradiol levels. Unfortunately, we did not determine progesterone levels in our study. However, progesterone levels rise during a long agonist IVF stimulation, which might affect appetite and food intake [17, 18].

It has been shown that leptin is necessary for human reproduction since starvation-induced leptin suppression and genetic models of leptin deficiency are associated with hypogonadism due to hypothalamic GnRH deficiency [19]. Leptin also modulates steroidogenesis in human ovaries [19]. In our study, the secretion of GnRH was blocked first, and then exogenic FSH was used, which induced a rise in estradiol levels and also an increase in leptin levels; hence our findings were similar. Results about leptin levels during the menstrual cycle are controversial, but some studies have reported a slight increase in leptin during the late follicular phase compared with the early follicular phase. This finding agrees with our results on leptin [20, 21]. According to a comprehensive review article, most studies have found a significant alteration in leptin concentrations across the menstrual cycle [22]. In addition, it has been demonstrated that leptin levels remained relatively constant for two hours postprandially in lean subjects and decreased gradually over the first 90 min in obese subjects [23]. Our results were different, with the most notable decrease between 30 and 60 min in Visit 2 and between 0 and 30 min in Visit 3. We did not separate obese and lean subjects, and the median BMI was 25.3. Furthermore, leptin induces sympathetic overactivity, particularly in people with obesity, which results in enhanced energy expenditure due to elevated epinephrine release at high estradiol levels [24, 25]. This phenomenon prevents weight gain and may explain our results about unchanging weight during the stimulation.

GLP-1 is an anorexigenic hormone, and it increases insulin secretion from the pancreatic b-cells, satiety and suppresses appetite [7, 8]. GLP-1 and insulin levels remained stable in our study, and energy intake diminished during the stimulation while rising estradiol levels. Evidence about GLP-1's stimulatory impact on a hypothalamic–pituitary level of the reproductive axis in animals exists [19]. GnRH-agonist is used in a long agonist protocol, and the secretion of endogenic FSH and LH is blocked, and then exogenic FSH is used. The reason why GLP-1 levels were unchanged in our study remains unclear. Also, no change in GLP-1 levels occurred postprandially, which differs from the previous results. It has been shown that GLP-1 levels rise quickly (10 min) postprandially [7, 23]. On the other hand, leptin has been shown to interact with GLP-1 and its receptor antagonist to induce satiety [9]. Leptin and GLP-1 seem to play an important role together in modulating appetite [9]. However, in our study, leptin levels peaked during the high estradiol level, whereas GLP-1 remained.

Toward the end of stimulation, while estradiol levels rose, proinflammatory factor hs-CRP diminished. This result conflicts with previous findings [26, 27]. Those studies concluded that controlled ovarian stimulation induces a state of systemic inflammation. An inflammatory response may also be stimulated by IVF-related treatments [28, 29]. Our findings are, however, contrary.

Leptin correlated significantly with insulin, lipids, weight, and hs-CRP in our study. Leptin has a similar anorexigenic effect than insulin has, and it is involved with insulin signaling, which explains the positive correlation [1, 5]. Leptin levels are associated with a body fat mass, and during a weight gain, leptin levels rise [6]. These things explain the positive correlation between leptin and lipids or weight in our results. The positive correlation between leptin and hs-CRP remains unclear in our study. Hs-CRP correlated negatively with HDL cholesterol concentration and positively with triglyceride concentration and weight. As a marker of low-grade inflammation, the hs-CRP level is chronically elevated in people with obesity, which may explain the positive correlation. Hs-CRP also correlated negatively with insulin at Visit 3, and this finding remains unclear in our results. Fasting glucose levels decreased between Visits 2 and 3, and lipid profile improved during the stimulation, and these findings support our earlier results about the safety of IVF stimulation [11, 12].

The validity of this study can be considered good since all these patients underwent the same IVF protocol in one unit, and they served as their controls. All laboratory tests and data about appetite profile and dietary intake were blindly analyzed by personnel who had no information of the IVF cycle phase. The diet was reported with eating diaries three days before each visit, hence the impact of a long agonist protocol on dietary intake could be examined. The cause of underlying infertility (for example, obesity, polycystic ovaries syndrome) may play a role in appetite regulation. A limitation of this study is the relatively small study population; limited statistical power in the analyses can affect our results.

## Conclusions

We found that, during a long agonist IVF protocol, when estradiol levels were rising, leptin concentrations increased, energy intake decreased, and weight remained stable. Thus, leptin may protect from overeating during high estradiol levels, and leptin resistance may not occur during a short period. Also, fasting glucose decreased, and lipid profile improved during the high estradiol levels. In addition, hs-CRP concentrations diminished while estradiol levels increased. Thus, an anti-inflammatory response was detected in our study. All these metabolic changes could be considered favorable regarding the possible IVF pregnancy. These results, similar to our previous findings, support the safety of IVF stimulation.

## Data Availability

The datasets generated and analyzed during the current study are not publicly available due to privacy protection but are available from the corresponding author on a reasonable request.

## Abbreviations

GLP-1: Glucagon-like peptide-1
hs-CRP: High-sensitivity C-reactive protein
IVF: In vitro fertilization
FSH: Follicle stimulating hormone
GnRH: Gonadotrophin-releasing hormone
BMI: Body mass index
NS: Non-significant
LDL: Low-density lipoprotein
HDL: High-density lipoprotein
SD: Standard deviation
SEM: Standard error of mean
